# Blends of Furanosesquiterpenes from *Smyrnium olusatrum*: An Alternative Strategy for Mosquito Control

**DOI:** 10.3390/plants15213485

**Published:** 2026-11-08

**Authors:** Giada Trebaiocchi, Eleonora Spinozzi, Roman Pavela, Loredana Cappellacci, Filippo Maggi, Riccardo Petrelli

**Affiliations:** 1 Chemistry Interdisciplinary Project (ChIP) Research Center, School of Pharmacy, University of Camerino, Via Madonna delle Carceri, 62032 Camerino, Italy; giada.trebaiocchi@unicam.it (G.T.); loredana.cappellacci@unicam.it (L.C.); filippo.maggi@unicam.it (F.M.); riccardo.petrelli@unicam.it (R.P.); 2 Czech Agrifood Research Center, Dunovská 507, Ruzyně, 161 06 Prague, Czech Republic; roman.pavela@carc.cz; 3 Department of Plant Protection, Faculty of Agrobiology, Food and Natural Resources, Czech University of Life Sciences Prague, Kamycká 129, Suchdol, 165 00 Prague, Czech Republic; 4 Department of Plant Biotechnology, College of Life Sciences and Biotechnology, Korea University, Seoul 02841, Republic of Korea

**Keywords:** furanosesquiterpenes, *Smyrnium olusatrum*, *Culex quinquefasciatus*, larvicidal, synergism, non-target

## Abstract

The recent outbreak of vector-borne diseases has highlighted the crucial importance of effective mosquito control programs. Nevertheless, mosquito management still represents a challenge due to the rapid onset of resistance to conventional insecticides. In this regard, there is an increasing research interest in botanicals as attracting valuable sources of insecticidal compounds. *Smyrnium olusatrum* L. (Apiaceae), an aromatic plant of great scientific relevance, owes its bioactivity to furanosesquiterpenes, whose major constituents include isofuranodiene (IFD), curzerene, 1-*β*-acetoxyfuranoeudesm-4(15)-ene, and atractylon. Since these compounds have demonstrated promising larvicidal activity against different mosquito species, in this study they were tested individually and in blends against *Culex quinquefasciatus* Say larvae, demonstrating the occurrence of synergistic interactions leading to enhanced larvicidal activity. In detail, the blend (1:1) consisting of curzerene and atractylon displayed the highest synergistic action (61.25% mortality), double with respect to that expected (33.75%). Moreover, the pure compounds were tested at their LC_50_ concentrations against the non-target species *Daphnia magna* Straus, *Chaoborus flavicans* Meigen, and *Tubifex tubifex* Müller, with mortalities from 7.5 to 33.7%. Overall, these findings demonstrate that synergistic furanosesquiterpene blends represent a promising botanical strategy for mosquito control, while minimizing the environmental impact associated with conventional insecticides.

## 1. Introduction

In recent years, vector-borne diseases (VBDs), particularly those transmitted by mosquitoes, have been a cause of global health concern [1], highlighting the crucial importance of developing effective mosquito control programs. Nowadays, the use of synthetic insecticides remains the main approach for vector control. However, the latter caused the onset of adverse effects on human health, environment, and non-target organisms. In addition, the extensive use of these insecticides led to the development of insecticide resistance and thus to a lack of efficacy [2,3]. In many cases, the onset of resistance is associated with the use of a single active compound, against which mosquitoes can develop detoxification and sequestration strategies, rendering the insecticidal compound ineffective [4].

In this scenario, various botanical-based products have been reported for their considerable potential in vector management, as they are target-specific, safer, and less toxic than conventional insecticides, in addition to being naturally biodegradable and cheap [5,6]. Furthermore, plant-derived compounds have shown increased activity and lower toxicity when used in blends, highlighting their potential as sources of synergistic combinations with high effectiveness [7]. The latter is achieved through a multi-target effect to which the mosquito cannot adapt [8]. For the above-mentioned reasons, there is a significant interest in plant-derived blends with insecticidal potential, since they are often encompassed with improved bioactivity, wider spectrum of action, longer safety, lower risks related to insect resistance and environmental impact [7,9].

Among the botanicals employable as insecticides, furanosesquiterpenes have proven promising potential for their larvicidal activity on different mosquito species [10,11]. A rich plant source of furanosesquiterpenes is *Smyrnium olusatrum* L. (Apiaceae), a stout, glabrous, celery-scented biennial herb up to 150-200 cm tall. It is endowed with secretory structures, characterized by high uniformity in distribution and content. These structures are the main site of production of the essential oil (EO) since they are filled with a secretion containing lipophilic volatile compounds, mainly represented by furanosesquiterpenes [12]. *S. olusatrum* is distributed in different countries with varying humidity and temperature levels such as Mediterranean regions, Western Asia, Southern Europe and North African countries, yielding EOs with various chemical compositions [13]. The plant is widely known for its extensive use in both food and traditional medicine and it possesses many bioactivities, such as antioxidant, antibacterial, antifungal, insecticidal, hepatoprotective, apoptotic, antibacterial, and anticancer [13]. As reported by Benelli et al. [14], the EO resulted effective against *C. quinquefasciatus* larvae, with an LC_50_ of 17.5 μL/L [14].The main furanosesquiterpenes found in the EO are isofuranodiene, curzerene, 1-*β*-acetoxyfuranoeudesm-4(15)-ene, and, to a minor extent, atractylon. Regarding these compounds, a promising larvicidal activity has been observed against different mosquito species [15].

For instance, Benelli et al. [11] and Pavela et al. [16] reported the larvicidal activity of isofuranodiene against 3^rd^ instar larvae of *Culex quinquefasciatus* Say with an LC_50_ lower than 34 µg/mL. The same compound has also been tested on *Aedes aegypti* L. with an LC_50_ of 5.58 µg/mL. On the other hand, also curzerene was tested on *Ae. aegypti* showing a LC_50_ value of 7.44 µg/mL [10]. The activity of this compound was also evaluated against *Aedes albopictus* Skuse (LC_50_ of 6.74 µg/mL), also confirming in this case its larvicidal potential [17]. Regarding atractylon, limited data on its larvicidal potential on mosquitoes are available. Nevertheless, it has shown a LD_50_ value of 1.43 µg/adult on the fruit fly *Drosophila melanogaster* Meigen [18].

The objective of this study was to evaluate for the first time the larvicidal activity of blends of the main furanosesquiterpenes found in *S. olusatrum* on *C. quinquefasciatus* larvae and to assess their possible synergistic interaction. Simultaneously, the effects of these compounds were evaluated on the non-target aquatic species *Daphnia magna* Straus, *Chaoborus flavicans* Meigen, and *Tubifex tubifex* Müller to assess their potential impact on highly sensitive aquatic organisms.

## 2. Results

### 2.1. Chemical Analysis of the Essential Oil (EO) of Smyrnium olusatrum

The EO of *S. olusatrum* -used as a reference product derived from *S. olusatrum* with proven larvicidal activity-was analyzed via qualitative GC-MS, and its detailed chemical characterization is presented in Table S1 (Supplementary Materials). The EO chemical profile (total identified 96.72%) was mainly dominated by furanosesquiterpenes (60.06%), followed by monoterpene hydrocarbons (21.14%) and sesquiterpene hydrocarbons (15.44%). Among furanosesquiterpenes, 1-*β*-acetoxyfuranoeudesm-4(15)-ene (28.40%), curzerene (17.04%), and isofuranodiene (11.17%) resulted the predominant compounds. Conversely, *β*-phellandrene and *α*-pinene were the main monoterpene hydrocarbons found (9.43 and 6.55%, respectively). As regards sesquiterpene hydrocarbons, germacrene D was the main representative of this chemical class (9.63%). The relative abundance was calculated for each identified compound and expressed as a percentage of the total peak area of the detected compounds. The analyses were performed in triplicate (*n* = 3).

### 2.2. Isolation and Synthesis of Furanosesquiterpenes

The furanosesquiterpenes employed in this study were isolated from *S. olusatrum* EO when present, while the remaining compounds were synthetized. Their chemical characterization is herein reported, while their NMR spectra are reported in the Supplementary Materials (Figure S1–S8).


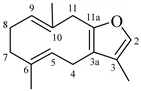


*Isofuranodiene, (5E,9Z)-3,6,10-trimethyl-4,7,8,11-tetrahydrocyclodeca[b]furan.* White solid (77% yield). IR (neat) 2964, 2924, 2861, 1664, 1437, 1386, 1138, 754 cm^−1^; ^1^H NMR (500 MHz, DMSO) *δ* 7.24 (s, 1H, H-2), 4.98–4.91 (m, 1H, H-9), 4.72–4.62 (m, 1H, H-5), 3.47–3.38 (m, 2H, H-11), 3.05 (dt, *J* = 29.4, 13.5 Hz, 2H, H-4), 2.20 (dt, *J* = 11.8, 3.0 Hz, 1H, H-8), 2.12–2.04 (m, 2H, H-7), 1.88 (d, *J* = 1.2 Hz, 3H, 3-methyl), 1.73 (ddd, *J* = 26.6, 13.2, 7.4 Hz, 1H, H-8), 1.57 (s, 3H, 10-methyl), 1.21 (s, 3H, 6-methyl); ^13^C NMR (126 MHz, DMSO) *δ* 148.79 (C-11a), 136.11 (C-2), 133.45 (C-10), 128.83 (C-6), 128.31 (C-9), 127.20 (C-5), 121.37 (C-3), 118.55 (C-3a), 26.34 (C-11), 23.73 (C-7), 16.19 (C-8, C-4), 15.95 (6-Me, 10-methyl), 8.52 (3-methyl); MS (EI): *m/z* = 216 (M^+^), 159, 145, 108, 93; Anal. calcd. for C_15_H_20_O: C, 83.28; H, 9.32; found: C, 83.25; H, 9.34. The chemical characterization of isofuranodiene was linear with that previously reported by Pavela et al. [16].


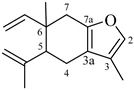


*Curzerene, 3,6-dimethyl-5-(prop-1-en-2-yl)-6-vinyl-4,5,6,7-tetrahydrobenzofuran.* Colorless oil (21% yield). IR (neat) 3082, 2968, 2908, 1637, 1441, 1414, 1112, 734 cm^−1^. ^1^H NMR (500 MHz, DMSO) *δ* 7.21 (s, 1H, H-2), 5.87 (dd, *J* = 17.5, 10.8 Hz, 1H, H2-vinyl), 5.01–4.93 (m, 2H, H1-propenyl, H1-vinyl), 4.85–4.81 (m, 1H, H1-vinyl), 4.75 (d, *J* = 1.3 Hz, 1H, H1-propenyl), 2.37–2.22 (m, 5H, H-4, H-7, H-5), 1.87 (d, *J* = 1.2 Hz, 3H, 3-methyl), 1.70 (d, *J* = 0.4 Hz, 3H, H3-propenyl), 1.00 (s, 3H, 6-methyl); ^13^C NMR (126 MHz, DMSO) δ 148.71 (C-7a), 146.85 (C2-propenyl), 146.64 (C2-vinyl), 137.31 (C-2), 118.74 (C-3, C-3a), 116.11 (C1-vinyl), 112.77 (C1-propenyl), 111.09 (C-5), 49.05 (C-6), 35.64 (C-7), 24.00 (C3-propenyl), 23.46 (6-methyl), 19.14 (C-4), 7.75 (3-methyl); MS (EI): *m/z* = 216 (M^+^), 148, 108, 201, 79; Anal. calcd. for C_15_H_20_O: C, 83.28; H, 9.32; found: C, 83.24; H, 9.31. The chemical characterization was linear to that previously reported [19].


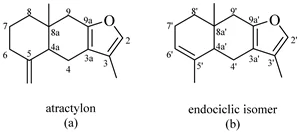


*Atractylon, 3,8a-dimethyl-5-methylene-4,4a,5,6,7,8,8a,9-octahydronaphtho[2,3-b]furan (a) and 3,5,8a-trimethyl-4,4a,7,8,8a,9-hexahydronaphtho[2,3-b]furan (b)*. Mixture of the exocyclic (a) (48.84%) and endocyclic (51.16%) (b) isomers [20,21], corresponding to a molar ratio of approximately 1:1.05. Colorless oil (67% yield). IR (neat) 2989, 2913, 2845, 1655, 1470, 1408, 1119, 738 cm^−1^. ^1^H NMR (500 MHz, DMSO) δ 7.20 (s, 2H, H-2 and H-2′), 5.41 (dq, *J* = 5.4, 2.0 Hz, 1H, H-6′), 4.85 (q, *J* = 1.8 Hz, 1H, H-methylene), 4.69 (q, *J* = 1.8 Hz, 1H, H-methylene), 2.56 (ddd, *J* = 15.6, 4.9, 1.6 Hz, 1H, H-4a), 2.46–2.38 (m, 1H, H-4a′), 2.39–2.18 (m, 6H, H-4, H-4′, and H-6), 2.14–1.91 (m, 6H, H-7′, H-9, and H-9′), 1.89 (dd, *J* = 2.3, 1.3 Hz, 6H, 3-methyl and 3′-methyl), 1.68 (dt, *J* = 2.7, 1.4 Hz, 3H, 5′-methyl), 1.64 (tt, *J* = 3.6, 1.7 Hz, 2H, H-7), 1.57–1.45 (m, 4H, H-8 and H-8′), 0.75 (s, 3H, 8a′-methyl), 0.68 (s, 3H, 8a-methyl). ^13^C NMR (126 MHz, DMSO) δ 149.42 (C-9a), 149.23 (C-9a′), 148.59 (C-5), 137.09 (C-2), 137.08 (C-2′), 133.99 (C-5′), 121.43 (C-6′), 119.98 (C-3), 118.95 (C-3′), 116.77 (C-3a), 115.72 (C-3a′), 107.39 (C-methylene), 44.97 (C-4a), 43.23 (C-4a′), 40.87 (C-9), 38.60 (C-9′), 38.05 (C-8a), 36.60 (C-8a′), 36.31 (C-6), 36.22 (C-7), 32.71 (C-7′), 23.05 (C-8), 22.22 (C-8′), 20.97 (3-methyl), 20.66 (3′-methyl), 20.29 (5′-methyl), 17.21 (C-4), 16.33 (C-4′), 7.85 (8a-methyl), 7.83 (8a′-methyl). MS (EI): *m*/*z* = 216 (M^+^), 201, 159, 145, 121, 108, 93, 91, 79. Anal. calcd. for C_15_H_20_O: C, 83.28; H, 9.32; found: C, 83.26; H, 9.36.


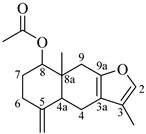


*1-β-Acetoxyfuranoeudesm-4(15)-ene, 3,8a-dimethyl-5-methylene-4,4a,5,6,7,8,8a,9-octahydronaphtho[2,3-b]furan-8-yl acetate*. Colorless oil (22.8% yield). IR (neat) 2982, 2812, 2765, 1728, 1646, 1467, 1398, 1243, 1123, 746 cm^−1^. ^1^H NMR (500 MHz, DMSO) δ 7.21 (s, 1H, H-2), 4.93 (d, *J* = 1.7 Hz, 1H, 5-methylene), 4.82 (dd, *J* = 11.9, 4.4 Hz, 1H, H-8), 4.78 (d, *J* = 1.9 Hz, 1H, 5-methylene), 2.47–2.11 (m, 7H, H-6, H-4a, H-4, H-9), 2.06 (s, 3H, H-acetyl),1.90 (d, *J* = 1.4 Hz, 3H, 3-methyl), 1.80 (dtd, *J* = 12.1, 4.7, 2.7 Hz, 1H, H-7), 1.51 (dtd, *J* = 13.7, 12.0, 4.4 Hz, 1H, H-7), 0.72 (s, 3H, 8a-methyl). ^13^C NMR (126 MHz, DMSO) δ 170.52 (C=O acetyl), 148.58 (C-9a), 147.45 (C-5), 137.93 (C-2), 119.41 (C-3), 115.80 (C-3a), 109.53 (5-methylene), 79.67 (C-8), 43.57 (C-4a), 35.55 (C-8a), 33.67 (C-9), 28.14 (C-6), 26.82 (C-7), 21.35 (C-4), 20.39 (methyl-acetyl), 12.29 (3-methyl), 8.31 (8a-methyl). MS (EI): *m*/*z* = 274 (M^+^), 214.1, 199.1, 185.1, 172.1, 159, 145, 108, 91, 79. Anal. calcd. for C_17_H_22_O_3_: C, 74.04; H, 8.08; found: C, 74.10; H, 8.02.

### 2.3. Larvicidal Assays

#### 2.3.1. Acute Toxicity

The pure compounds, namely isofuranodiene, curzerene, atractylon, and 1-*β*-acetoxyfuranoeudesm-4(15)-ene, were tested on 3^rd^ instar larvae of *C. quinquefasciatus* to assess their acute toxicity before the evaluation of their synergistic interactions. Their toxicity was compared to that of the EO. As reported in Table 1, the efficacy of isofuranodiene and atractylon was comparable to that of the EO, with LC_50_ values of 9.8, 12.1, and 10.1 µL/L, respectively. Curzerene showed the lowest larvicidal activity (LC_50_ = 19.6 µL/L), whereas 1-*β*-acetoxyfuranoeudesm-4(15)-ene displayed an intermediate efficacy (LC_50_ = 15.2 µL/L) (Table 1).

#### 2.3.2. Synergistic Interactions

The aim of this work was to assess for the first time the potential interactions of the furanosesquiterpenes of *S. olusatrum* when tested in blends. The blend ratios were not intended to reproduce the EO composition. The EO was used solely as a reference product derived from *S. olusatrum* with previously demonstrated larvicidal activity. As specified in Section 4.4.2, the blends were tested at their sublethal concentration (4.0 µL/L), which allowed the evaluation of potential synergistic, antagonistic, or additive effects. In detail, a blend producing significantly higher or lower mortality than the most effective individual constituent was considered to exhibit a synergistic or antagonistic effect. When no significant difference was detected, the interaction was classified as additive. The blends were compared to the pure compounds tested alone as well as *S. olusatrum* EO, in order to compare them with a *S. olusatrum*-derived product already known for its larvicidal potential.

**Figure 1:**
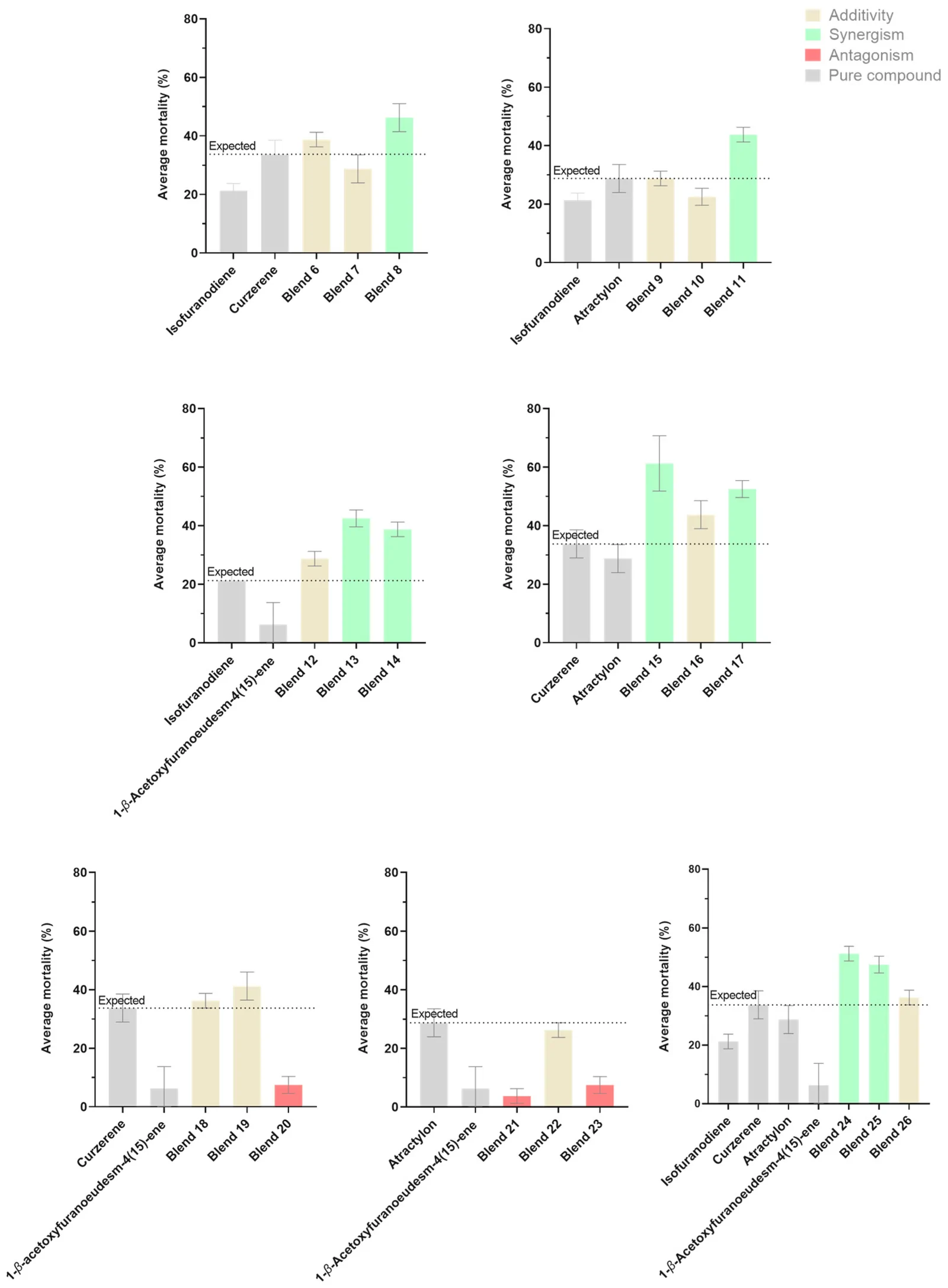
Graphical representation of the synergistic, antagonistic, or additive effects of the tested blends. The composition of each blend is reported in Table 2.

Concerning the binary blends of furanosesquiterpenes, a synergistic effect was detected for blends 8, 11, 13–15, and 17 (Table 2 and Figure 1). More specifically, blends with the highest effect were those containing curzerene, namely 15, 17, and 8, with mortalities of 61.25, 52.50, and 46.25%, respectively. Blend 15 (curzerene + atractylon, 1:1) was the most effective, even 6 times more effective (61.25% mortality) than the EO (11.75% mortality) (Table 2 and Figure 1). Conversely, blends 20, 21, and 23 were those among the binary blends exhibiting an antagonistic effect, with efficacies significantly lower (7.50, 3.75, and 7.50% mortality, respectively) than the most active compound of the blend. Interestingly, all those blends contained 1-*β*-acetoxyfuranoeudesm-4(15)-ene, which was also the less effective compound at sublethal doses (6.25% mortality).

As regards the ternary mixtures, blends 24 and 25 (isofuranodiene + curzerene + 1-*β*-acetoxyfuranoeudesm-4(15)-ene and isofuranodiene + curzerene + atractylon) resulted in a synergistic effect, with mortalities of 51.25 and 47.50%, respectively (Table 2 and Figure 1). All the other blends tested displayed a simple additive effect.

### 2.4. Toxicity to Non-Target Organisms

The evaluation of the interactions among furanosesquiterpenes when tested in blends was accompanied by an assessment of the toxicity of the pure compounds (isofuranodiene, curzerene, atractylon, and 1-*β*-acetoxyfuranoeudesm-4(15)-ene) on three non-target aquatic organisms, namely *D. magna*, *C. flavicans*, and *T. tubifex*. At concentrations corresponding to the LC_50_ for mosquito larvae, mortality of all three non-target organisms was 7.5–33.7% (Table 3).

Interestingly, the less toxic product at the tested concentration resulted curzerene (mortalities from 7.5 to 18.7%), followed by isofuranodiene (7.5–28.7% mortality). Moreover, the toxicity caused by curzerene was almost comparable to that of the EO.

Nevertheless, at concentrations corresponding to the LC_90_, every product resulted toxic (Table 4).

## 3. Discussion

The acute toxicity determined for pure compounds meets a commonly used screening criterion for promising candidates in the development of botanical larvicides since their LC_50_ resulted far below 100 µg/mL [22]. Our findings confirm the promising larvicidal activity already found for some of the furanosesquiterpenes tested in this work, especially isofuranodiene and curzerene. For instance, isofuranodiene has been previously tested on 3^rd^ instar larvae of *C. quinquefasciatus* as well as on *Ae. aegypti* with LC_50_ < 34 and of 5.58 µg/mL, respectively [10,14,16]. As concerns curzerene, this compound displayed a high efficacy on *Ae. aegypti* (LC_50_ of 7.44 µg/mL) and *Ae. albopictus* (LC_50_ of 6.74 µg/mL) [10,17]. On the other hand, our work reports for the first time the larvicidal activity of atractylon on *C. quinquefasciatus*. Indeed, the compound has been tested for its pesticidal potential only on the fruit fly *D. melanogaster* with a LD_50_ value of 1.43 µg/adult [18]. Moreover, this is the first time that the larvicidal potential of 1-*β*-acetoxyfuranoeudesm-4(15)-ene has been reported and this represents a starting point for the understanding of the larvicidal potential of this compound.

Nevertheless, botanical insecticides are generally applied as complex mixtures rather than as single purified constituents, and increasing evidence indicates that interactions among their components may markedly influence biological activity. Synergistic combinations can enhance larvicidal efficacy, broaden the spectrum of action, reduce the amount of active ingredient required, and potentially delay the evolution of insect resistance [7]. Therefore, evaluating the interactions among the furanosesquiterpenes of *S. olusatrum* is essential to determine how the activity of the EO, which has been extensively reported before, could be influenced by additive, antagonistic or synergistic effects.

Among the different blends tested, the most outstanding resulted that containing curzerene and atractylon at 1:1 ratio (blend 15) (Table 2). The high synergistic action (61.25% mortality) of this blend was almost double with respect to that expected (33.75% mortality) and six times higher than that of the EO, employed as reference. Other effective synergistic blends were 8, 11, 13, 14, and 17, all containing curzerene. This result was quite expected, since curzerene was the most effective compound at sublethal doses. This is fully consistent with the concept that mixtures of compounds can overcome their major single components due to intra mixture interactions. This synergistic activity of plant-derived compounds can be easily confirmed with other studies. For instance, Scalerandi et al. [23] demonstrated that mixtures of terpenes were more active than the same compounds tested alone. In detail, this study investigated the activity of mixtures of terpenes against *Musca domestica* L. (Diptera: Muscidae). Their activity was related the interactions among the compounds rather than to that of the most active compound [23].

Interestingly, the binary blends presenting an antagonistic effect among their constituents were blends 20, 21, and 23, with efficacy significantly lower (7.50, 3.75, and 7.50% mortality, respectively) than the most active compound of the blend. These blends all contained 1-*β*-acetoxyfuranoeudesm-4(15)-ene, which was also the least effective compound at sublethal doses (6.25% mortality) (Table 2 and Figure 1). These results suggest that 1-*β*-acetoxyfuranoeudesm-4(15)-ene could be the potential cause of the low activity of the blends and of the EO if compared to isofuranodiene and curzerene. The furanosesquiterpenes mode of action is not known, but their efficacy could be linked to their lipophilicity which allows easy penetration of the insect cuticle [16]. Then, the toxicity of these compounds could be given by their possible conversion into sesquiterpene lactones [24], although this hypothesis needs to be confirmed. Therefore, when tested in blends, furanosesquiterpenes might enhance their cuticle penetration, potentially contributing to the observed synergism. Conversely, 1-*β*-acetoxyfuranoeudesm-4(15)-ene might antagonize the bioactivity of the other compounds due to its lower lipophilicity. The hypothesis of synergism can be easily explained by some studies already reported in the literature [7]. For instance, a previous study reported the toxicity of *Rosmarinus officinalis* L. (Lamiaceae) EO on the cabbage looper *Trichoplusia ni* (Lepidoptera: Noctuidae). However, the pure major compounds of this EO could not achieve the insecticidal activity reported, while the synergistic action of the blend 1,8-cineole and camphor could explain the 90% of the bioactivity.

A similar effect was observed in the mode of action of two major constituents of *Thymus vulgaris* L. (Lamiaceae) EO, thymol and *p*-cymene. In this case, the latter compound synergized toxicity of the first one through increased penetration of thymol in adult housefy, *M. domestica* [7].

Overall, the relatively low mortality observed in the three non-target species indicates that the tested furanosesquiterpenes exhibit a favorable degree of selectivity toward mosquito larvae. Although *D. magna* appeared to be the most sensitive organism, none of the compounds caused mortality exceeding 33.7% at concentrations effective against the target species, supporting their potential as environmentally compatible botanical larvicides [25,26,27]. However, the assessment of their non-target toxicity at their LC_90_ values led to the complete mortality of the tested organisms. Nevertheless, this is the first time that furanosesquiterpenes were tested on *C. flavicans*, an important invertebrate predator, and *T. tubifex*, a worm widely employed for pesticide toxicity evaluation, thus furnishing important information on the safety of these compounds.

## 4. Materials and Methods

### 4.1. Chemicals and Instrumentation

The solvents employed for the synthesis and purification of furanosesquiterpenes as well as DMSO were purchased from Merck (Darmstadt, Germany). ^1^H (500 MHz) and ^13^C (126 MHz) spectra were acquired using a Bruker Avance III 500 MHz spectrometer (Bruker, Billerika, MA, USA) and dissolving the pure compounds in deuterated dimethylsulfoxide (DMSO) (Merck, Milan, Italy). The chemical shifts for ^1^H- and ^13^C-NMR are expressed as *δ* [parts per million (ppm)] while the coupling constants are reported in hertz. The couplings in the spectra are expressed with the following abbreviations: s (singlet), d (doublet), dd (doublet of doublets), ddd (doublet of doublets of doublets), t (triplet), dt (doublet of triplets), dtd (doublet of triplets of doublets), tt (triplet of triplets), q (quartet), m (multiplet). IR (cm^−1^) spectra were acquired using a Perkin-Elmer FT-IR spectrometer Spectrum Two UATR (Perkin Elmer, Inc., Waltham, MA, USA). Mass analyses were conducted by using GC-MS with an Agilent 8890 gas chromatograph furnished of a 5977B single quadrupole mass spectrometer, operating in electron impact (EI) mode (Agilent, Santa Clara, CA, USA) and a PAL RTC120 autosampler (CTC Analytics AG, Zwingen, Switzerland). Pure compounds were named and numbered following IUPAC rules as applied by ChemBioDraw Ultra (version 13.0) software for systematically naming organic compounds.

### 4.2. Essential Oil (EO) Extraction

The EO of *S. olusatrum* was of the same batch of that employed in our previous study and it was obtained by conventional hydrodistillation of *S. olusatrum* ripe fruits (EO yield 2.64%) [28]. This EO was employed only to compare the activity of furanosesquiterpenes blends to a well-known larvicidal product. The EO was analyzed by employing the GC-MS instrument described in Section 4.1. The analytical methodology as well as the identification of the compounds followed the work previously reported [28,29,30,31,32,33]. The relative abundance was calculated for each identified compound and expressed as a percentage of the total peak area of the detected compounds.

### 4.3. Furanosesquiterpenes Isolation and Synthesis

The isolation or synthesis of the furanosesquiterpenes was achieved by following different procedures and the compounds were all chemically characterized through NMR, GC-MS and IR analysis.

#### 4.3.1. Isolation of Isofuranodiene

Isofuranodiene was obtained from *S. olusatrum* EO through crystallization by dissolving 17.77 g of EO in methanol and by storing the solution at −20 °C. After precipitation, the crystals were filtered and washed with cold methanol to remove further impurities.

#### 4.3.2. Synthesis of Curzerene

Isofuranodiene (1.0 g) was dissolved in ethylene glycol (10 mL) and left under stirring under reflux for 90 min in an inert atmosphere. Once the reaction was completed, the mixture was cooled down to room temperature and then extracted with diethyl ether. The organic phase was dried over Na_2_SO_4_, filtered, and evaporated to dryness. The crude product was purified by silica gel column chromatography employing hexane as mobile phase.

#### 4.3.3. Synthesis of Atractylon

This synthesis leads to the formation of a mixture of atractylon endocyclic and exocyclic isomers [20,21]. Isofuranodiene (1.0 g) was dissolved in dichloromethane (216 mL) at 20 °C. Then, a catalytic amount of *p*-toluenesulfonic acid was added, and the reaction mixture was stirred for 60 min at the same temperature. At the end of the reaction, the solvent was evaporated to dryness under vacuum, and the mixture was further purified by silica gel column chromatography using hexane as mobile phase. As the individual endocyclic and exocyclic atractylon isomers could not be isolated despite repeated attempts, the mixture was characterized by NMR, GC-MS, and IR analyses and tested as such in the larvicidal assays, hereafter referred to as “atractylon”. The relative composition of the exocyclic (a) and endocyclic (b) atractylon isomers was estimated through the integration of diagnostic ^1^H-NMR resonances, according to the principles of NMR-based relative quantification described in the literature [34]. No internal standard or authentic reference compounds were required for this relative estimation.

#### 4.3.4. Isolation of 1-*β*-acetoxyfuranoeudesm-4(15)-ene

The compound was isolated from *S. olusatrum* EO. In detail, 1-*β*-acetoxyfuranoeudesm-4(15)-ene was purified by a gradient silica gel column chromatography starting with hexane and then increasing the polarity of the mobile phase (hexane/ethyl acetate, 99:1).

### 4.4. Larvicidal Assays

#### 4.4.1. Acute Toxicity

Acute toxicity, assessed as larval mortality 24 h after application of the tested substances, was evaluated on 3^rd^ instar larvae of the mosquito *C. quinquefasciatus* Say (Diptera: Culicidae) under laboratory conditions. Larvae were obtained from a long-term laboratory colony (>20 generations; Czech Agrifood Research Center, Czech Republic). Larvae were fed sourdough bread; adults were provided with a 10% sucrose solution, and after mating, females were blood-fed on laboratory mice. Mosquito rearing and all experiments described below were conducted at 25 ± 2 °C, 70 ± 5% relative humidity, and a 16:8 h (light:dark) photoperiod.

Acute toxicity assays followed WHO [35] standard procedures with minor modifications. The tested furanosesquiterpenes were first dissolved in DMSO at a 1:1 (*v*/*v*) ratio to prepare stock solutions. These stock solutions were subsequently diluted with water to obtain a concentration series eliciting larval mortality of approximately 10–90% (range estimated from preliminary experiments). The tested concentrations were 3.0, 5.0, 8.0, 10.0, 12.0, 15.0, 20.0, 25.0, 30.0, 35.0, and 40.0 µL/L. DMSO at 20.0 µL/L served as the solvent control.

Each dilution was prepared in 200 mL of water in a glass beaker (water surface area 125 cm^2^). Twenty 3^rd^ instar larvae were transferred into each beaker; four replicates were performed for each concentration. Larvae were not fed during the 24 h exposure. Mortality was assessed after 24 h; larvae were considered dead if they did not respond with a clear movement to gentle mechanical stimulation.

#### 4.4.2. Synergistic Interactions

The synergistic interactions were tested using the same procedure as for acute larval toxicity, except that stock solutions of blends of pure compounds were first prepared at various experimental ratios (Table 5).

To investigate the interaction potential of the furanosesquiterpenes, blends were tested only at the sublethal concentration of 4.0 µL/L, which, based on preliminary tests, was expected to produce 10–30% mortality and, consequently, to allow potential synergistic or antagonistic effects among compounds to be detected. The mortality caused by each blend was compared with that caused by its individual constituent compounds tested at the same concentration. Moreover, their activity was compared to that of the EO, employed just as reference larvicidal product.

To evaluate the interactions among the constituents of the blends, a methodology based on an effect-based strategy was adopted. Specifically, this methodology is employed also in pharmaceutical studies and compares the effect originating from the combination of two or more compounds to the effects of the individual components [36,37]. Then, the evaluation of a synergistic, antagonistic, or additive effect was based on an approach also known as the Gaddum’s noninteraction or cooperative effect [38,39]. So, the presence of synergistic interaction was assumed when the blend produced a significantly higher mortality than the most effective individual constituent (Tukey’s HSD test, *p* < 0.01). Conversely, a blend producing significantly lower mortality (Tukey’s HSD test, *p* < 0.01) than the most effective constituent was considered antagonistic. When no significant difference was detected, the interaction was classified as additive.

All experiments were performed in four independent replicates under controlled environmental conditions in an air-conditioned chamber. The temperature was maintained at 25 ± 1 °C, the relative humidity at 70 ± 5%, and the photoperiod was set to 16:8 h (light:dark).

### 4.5. Toxicity to Non-Target Aquatic Organisms

Adults (2–5 days old) of *D. magna* used in the assays were obtained from a long-term laboratory colony (>20 generations; Czech Agrifood Research Center, Czech Republic). Adult and juvenile *D. magna* were reared in 10 L tanks and fed a yeast powder solution. 3^rd^ Instar larvae of *C. flavicans* and sexually mature adults of *T. tubifex* were purchased from a specialized supplier of live fish food (Habeo, Czech Republic). *C. flavicans* and *T. tubifex* were acclimated for one day to the experimental conditions and were supplied with dog biscuits as food.

The experiment followed the method of a previous work [40]. To assess the effects of the tested compounds on non-target aquatic organisms, individuals of *D. magna*, *C. flavicans*, and *T. tubifex* were exposed to concentrations corresponding to the LC_50_ and LC_90_ values previously determined for *C. quinquefasciatus* larvae.

Twenty individuals of *D. magna*, *C. flavicans*, or *T. tubifex* were introduced into plastic dishes containing 100 mL of water with the appropriate compound concentration. Mortality was determined after 48 h of exposure. Individuals showing no movement after gentle prodding with a fine brush were considered dead.

All experiments were performed in four independent replicates per treatment under controlled environmental conditions in an air-conditioned chamber. The temperature was maintained at 25 ± 1 °C, the relative humidity at 70 ± 5%, and the photoperiod was set to 16:8 h (light:dark).

### 4.6. Statistical Analysis

Control mortality between 1% and 20% was used to correct observed mortality using Abbott’s correction [41]. If control mortality exceeded 20%, experiments were discarded and repeated. LC_50_ and LC_90_ values and related parameters were estimated by probit analysis [42]. Mortality data (%), including tests of synergistic larvicidal potential and mortality of non-target species, were arcsine square root transformed prior to analysis, and significant differences were assessed by one-way ANOVA, followed by Tukey’s HSD multiple comparisons test (*p* < 0.01).

## 5. Conclusions

In the complex scenario of mosquito control, the research interest in innovative products is increasing and botanicals has been proven to be promising alternative agents. This work demonstrated for the first time synergistic interactions among the main furanosesquiterpenes found in *S. olusatrum* when tested in blends on *C. quinquefasciatus*. Blends containing curzerene, the most effective compound at sublethal doses, were the most outstanding. Among them, curzerene and atractylon demonstrated a synergistic action double with respect to that expected, suggesting their possible employment for mosquito control. Furthermore, we evaluated the non-target toxicity of the pure compounds on three non-target organisms, demonstrating that the toxicity was dose dependent. The promising synergistic activity observed for some blends could favor their potential application for the development of novel insecticidal products. Moreover, the limited toxicity detected for pure compounds, when tested at low doses, supports further investigations on their real-world applicability.

## Figures and Tables

**Table 1:** Larvicidal efficacy against *Culex quinquefasciatus*.

No.	Product Tested	LC_50_ (µL/L)	CI_95_	LC_90_ (µL/L)	CI_95_	Chi	*p*-Level	Df
1	EO	10.1	8.7–11.8	26.3	20.7–32.3	1.665	0.645	3
2	Isofuranodiene	9.8	8.7–10.9	35.4	27.7–42.8	2.191	0.533	3
3	Curzerene	19.6	17.8–24.3	39.7	30.9–61.2	0.222	0.973	3
4	Atractylon	12.1	7.1–16.2	59.8	37.9–84.3	0.193	0.978	3
5	1-*β*-Acetoxyfuranoeudesm-4(15)-ene	15.2	12.9–18.9	41.3	38.7–52.9	0.69	0.876	3

EO, essential oil.

**Table 2:** Evaluation of the interactions of furanosesquiterpenes when tested in blends on *Culex quinquefasciatus* larvae.

No.	Product Tested	Experimental Ratio ^a^	Expected Mortality (%) ^b^	Achieved Mortality ± SD (%) ^c^	Effect ^d^
1	EO ^e^	-	-	11.75 ± 2.36	-
2	Isofuranodiene	-	-	21.25 ± 2.50	-
3	Curzerene	-	-	33.75 ± 4.79	-
4	Atractylon	-	-	28.75 ± 4.79	-
5	1-*β*-Acetoxyfuranoeudesm-4(15)-ene	-	-	6.25 ± 7.50	-
6	Isofuranodiene + curzerene	1:1	33.75 ± 4.15	38.75 ± 2.50	additivity
7	Isofuranodiene + curzerene	2:1		28.75 ± 4.79	additivity
8	Isofuranodiene + curzerene	1:2		46.25 ± 4.79	synergism
9	Isofuranodiene + atractylon	1:1	28.75 ± 4.15	28.75 ± 2.50	additivity
10	Isofuranodiene + atractylon	2:1		22.50 ± 2.89	additivity
11	Isofuranodiene + atractylon	1:2		43.75 ± 2.50	synergism
12	Isofuranodiene + 1-*β*-acetoxyfuranoeudesm-4(15)-ene	1:1	21.25 ± 2.16	28.75 ± 2.50	additivity
13	Isofuranodiene + 1-*β*-acetoxyfuranoeudesm-4(15)-ene	2:1		42.50 ± 2.89	synergism
14	Isofuranodiene + 1-*β*-acetoxyfuranoeudesm-4(15)-ene	1:2		38.75 ± 2.50	synergism
15	Curzerene + atractylon	1:1	33.75 ± 4.15	61.25 ± 9.46	synergism
16	Curzerene + atractylon	2:1		43.75 ± 4.79	additivity
17	Curzerene + atractylon	1:2		52.50 ± 2.89	synergism
18	Curzerene + 1-*β*-acetoxyfuranoeudesm-4(15)-ene	1:1	33.75 ± 4.15	36.25 ± 2.50	additivity
19	Curzerene + 1-*β*-acetoxyfuranoeudesm-4(15)-ene	2:1		41.25 ± 4.79	additivity
20	Curzerene + 1-*β*-acetoxyfuranoeudesm-4(15)-ene	1:2		7.50 ± 2.89	antagonism
21	Atractylon + 1-*β*-acetoxyfuranoeudesm-4(15)-ene	1:1	28.75 ± 4.15	3.75 ± 2.50	antagonism
22	Atractylon + 1-*β*-acetoxyfuranoeudesm-4(15)-ene	2:1		26.25 ± 2.50	additivity
23	Atractylon + 1-*β*-acetoxyfuranoeudesm-4(15)-ene	1:2		7.50 ± 2.89	antagonism
24	Isofuranodiene + curzerene + 1-*β*-acetoxyfuranoeudesm-4(15)-ene	1:1:1	33.75 ± 4.15	51.25 ± 2.50	synergism
25	Isofuranodiene + curzerene + atractylon	1:1:1		47.50 ± 2.89	synergism
26	Isofuranodiene + curzerene + atractylon + 1-*β*-acetoxyfuranoeudesm-4(15)-ene	1:1:1:1		36.25 ± 2.50	additivity

^a^ Experimental ratio established for the test. ^b^ The expected mortality corresponds to the mortality caused by the most effective individual compound of the blend when tested alone. ^c^ Average achieved mortality calculated among four independent experiments, SD, standard deviation. ^d^ A mixture that caused significantly higher mortality (Tukey’s test, *p* < 0.01) than the most effective individual compound contained in it was considered to have a synergistic effect. Conversely, a mixture that caused significantly lower mortality (Tukey’s test, *p* < 0.01) than the most effective individual compound contained in it was considered to have antagonistic effect. A mixture that caused no significantly different mortality (Tukey’s test, *p* < 0.01) than the most effective individual compound contained in it was considered to have additive effect. ^e^ EO, essential oil.

**Table 3:** Mortality (%) of non-target organisms after application of concentrations corresponding to LC_50_ on mosquito larvae.

	Mortality ± SD (%) ^a^		
Product Tested	*Daphnia magna*	*Chaoborus flavicans*	*Tubifex tubifex*
EO ^b^	10.0 ± 3.5 bc	7.5 ± 2.5 b	0.0 ± 0.0 a
Isofuranodiene	28.7 ± 4.1 d	7.5 ± 2.5 b	18.7 ± 5.4 c
Curzerene	18.7 ± 4.1 c	7.5 ± 2.5 b	7.5 ± 2.5 b
Atractylon	33.7 ± 4.1 d	18.7 ± 4.1 c	10.0 ± 3.5 bc
1-*β*-Acetoxyfuranoeudesm-4(15)-ene	33.7 ± 4.1 d	11.3 ± 4.1 bc	13.8 ± 2.1 c
Negative control	0.0 ± 0.0 a	0.0 ± 0.0 a	7.5 ± 2.5 b
ANOVA_df5,18_ *F*; *p*-level	197.12; 0.000	87.65; 0.000	134.57; 0.000

^a^ Average achieved mortality calculated among four independent experiments, SD, standard deviation. ^b^ EO, essential oil. Different letters if present represent significant differences among mortalities.

**Table 4:** Mortality (%) of non-target organisms after application of concentrations corresponding to LC_90_ on mosquito larvae.

	Mortality ± SD (%) ^a^		
Product Tested	*Daphnia magna*	*Chaoborus flavicans*	*Tubifex tubifex*
EO ^b^	100.0 ± 0.0 c	82.5 ± 2.2 d	88.7 ± 3.7 c
Isofuranodiene	100.0 ± 0.0 c	61.2 ± 5.8 c	100.0 ± 0.0 d
Curzerene	96.2 ± 3.7 b	43.7 ± 9.1 b	42.5 ± 2.2 b
Atractylon	100.0 ± 0.0 c	83.7 ± 1.9 d	93.7 ± 3.7 c
1-*β*-Acetoxyfuranoeudesm-4(15)-ene	100.0 ± 0.0 c	81.2 ± 8.8 d	92.5 ± 5.0 c
Negative control	0.0 ± 0.0 a	0.0 ± 0.0 a	0.0 ± 0.0 a
ANOVA_df5,18_ *F*; *p*-level	1525.3; 0.000	387.52; 0.000	1201.45; 0.000

^a^ Average achieved mortality calculated among four independent experiments, SD, standard deviation. ^b^ EO, essential oil. Different letters if present represent significant differences among mortalities.

**Table 5:** Product tested for the synergistic effects evaluation on *Culex quinquefasciatus* larvae.

No.	Product Tested	Experimental Ratio
1	EO	-
2	Isofuranodiene	-
3	Curzerene	-
4	Atractylon	-
5	1-*β*-Acetoxyfuranoeudesm-4(15)-ene	-
6	Isofuranodiene + curzerene	1:1
7	Isofuranodiene + curzerene	2:1
8	Isofuranodiene + curzerene	1:2
9	Isofuranodiene + atractylon	1:1
10	Isofuranodiene + atractylon	2:1
11	Isofuranodiene + atractylon	1:2
12	Isofuranodiene + 1-*β*-acetoxyfuranoeudesm-4(15)-ene	1:1
13	Isofuranodiene + 1-*β*-acetoxyfuranoeudesm-4(15)-ene	2:1
14	Isofuranodiene + 1-*β*-acetoxyfuranoeudesm-4(15)-ene	1:2
15	Curzerene + atractylon	1:1
16	Curzerene + atractylon	2:1
17	Curzerene + atractylon	1:2
18	Curzerene + 1-*β*-acetoxyfuranoeudesm-4(15)-ene	1:1
19	Curzerene + 1-*β*-acetoxyfuranoeudesm-4(15)-ene	2:1
20	Curzerene + 1-*β*-acetoxyfuranoeudesm-4(15)-ene	1:2
21	Atractylon + 1-*β*-acetoxyfuranoeudesm-4(15)-ene	1:1
22	Atractylon + 1-*β*-acetoxyfuranoeudesm-4(15)-ene	2:1
23	Atractylon + 1-*β*-acetoxyfuranoeudesm-4(15)-ene	1:2
24	Isofuranodiene + curzerene + 1-*β*-acetoxyfuranoeudesm-4(15)-ene	1:1:1
25	Isofuranodiene + curzerene + atractylon	1:1:1
26	Isofuranodiene + curzerene + atractylon + 1-*β*-acetoxyfuranoeudesm-4(15)-ene	1:1:1:1

EO, essential oil.

## Data Availability

Data is contained within the article or Supplementary Materials.

## Supplementary Materials

The following supporting information can be downloaded at: https://www.mdpi.com/article/10.3390/plants15213485/s1, Table S1: Chemical composition of *Smyrnium olusatrum* EO; Figure S1: ^1^H NMR spectrum of isofuranodiene; Figure S2: ^13^C NMR spectrum of isofuranodiene; Figure S3: ^1^H NMR spectrum of curzerene; Figure S4: ^13^C NMR spectrum of curzerene; Figure S5: ^1^H NMR spectrum of atractylon; Figure S6: ^13^C NMR spectrum of atractylon; Figure S7: ^1^H NMR spectrum of 1-*β*-acetoxyfuranoeudesm-4(15)-ene; Figure S8: ^13^C NMR spectrum of 1-*β*-acetoxyfuranoeudesm-4(15)-ene. References [29,30,31,32,33] are cited in the supplementary materials.

## Abbreviations

The following abbreviations are used in this manuscript:

VBDs	Vector-Borne Diseases
EO	Essential Oil
DMSO	Dimethyl sulfoxide
