# Supplementary material for: Blends of Furanosesquiterpenes from Smyrnium olusatrum: An Alternative Strategy for Mosquito Control
Source: Plants (Basel). 2026 Nov 8;15(21):3485. doi: 10.3390/plants15213485 (PMC13765639; doi:10.3390/plants15213485)
Supplement: Supplementary file 1 [file plants-15-03485-s001.zip › plants-4641446-supplementary.pdf]

Supplementary Materials

Table S1. Chemical composition of *Smyrnium olusatrum* essential oil (EO).

No[a]	Compound	RI[b]	RI Lit[c]	Average ± SD %[d]	Id[e]
1	α-Thujene	925	924	0.17 ± 0.01	RI, MS
2	α-Pinene	931	932	6.55 ± 0.02	Std, RI, MS
3	Sabinene	971	969	0.16 ± 0.02	Std, RI, MS
4	β-Pinene	974	974	2.01 ± 0.07	Std, RI, MS
5	Myrcene	991	988	0.78 ± 0.04	Std, RI, MS
6	α-Phellandrene	1003	1002	0.33 ± 0.01	Std, RI, MS
7	δ-3-Carene	1009	1008	1.31± 0.01	RI, MS
8	α-Terpinene	1015	1014	0.03 ± 0.00	Std, RI, MS
9	ρ-Cymene	1023	1020	0.04 ± 0.01	Std, RI, MS
10	β-Phellandrene	1027	1025	9.43 ± 0.44	RI, MS
11	(*E*)-β-Ocimene	1048	1044	0.01 ± 0.00	Std, RI, MS
12	γ-Terpinene	1058	1054	0.04 ± 0.00	Std, RI, MS
14	Terpinolene	1087	1086	0.24 ± 0.01	Std, RI, MS
15	δ-Elemene	1337	1335	0.06 ± 0.01	Std, RI, MS
16	α-Copaene	1374	1374	0.05 ± 0.01	RI, MS
17	β-Elemene	1391	1389	1.92 ± 0.12	Std, RI, MS
18	(*E*)-Caryophyllene	1417	1417	0.07 ± 0.03	Std, RI, MS
19	α-Humulene	1452	1452	0.13 ± 0.03	Std, RI, MS
20	Germacrene D	1480	1484	9.63 ± 0.03	Std, RI, MS
21	Curzerene	1496	1499	17.04 ± 0.62	Std, RI, MS
22	γ-Cadinene	1513	1513	0.04 ± 0.02	Std, RI, MS
23	δ-Cadinene	1523	1522	0.70 ± 0.06	RI, MS
24	Germacrene B	1555	1559	2.91 ± 0.11	RI, MS
25	Isofuranodiene	1691	1679[f]	11.17 ± 0.38[h]	Std, MS
26	Germacrone	1694	1693	0.08 ± 0.02	RI, MS
27	Furanoeudesm-4(15)-en-1-one	1835	1833[g]	0.74 ± 0.17	RI, MS
28	Furanoeremophil-1-one	1900	1879[g]	0.11 ± 0.05	RI, MS
29	Glechomafuran	1925	1925[g]	1.61 ± 0.16	RI, MS
30	1-β-Acetoxyfuranoeudesm-4(15)-ene	1988	1988[g]	28.40 ± 0.44	RI, MS
31	1-β-Acetoxyfuranoeudesm-3-ene	1998	1998[g]	1.00 ± 0.13	RI, MS
					RI, MS
	Total identified (%)			96.72 ± 0.77	
	Monoterpene hydrocarbons			21.14	
	Sesquiterpene hydrocarbons			15.44	
	Oxygenated sesquiterpenes			0.08	
	Furanosesquiterpenes			60.06	

[a]Components are ordered according to their elution from the HP-5MS column. [b]Linear retention index calculated according to the Van den Dool and Kratz formula [29]. [c]Retention index taken from Adams. [d]Relative percentage values are mean of three independent analyses; SD, standard deviation. [e]Identification methods: Std, comparison with available analytical standard; RI, coherence of the calculated RI with those stored in the ADAMS (2007) [30] and NIST 17 (2017) [31] libraries; MS, mass spectrum matching with respect to ADAMS, and NIST 17 MS libraries. [f]RI reported by Papaioannou et al. [32]. [g]RI reported by Maggi et al. [33]. [h]The relative abundances of isofuranodiene and curzerene does not take into account the Cope rearrangement process, which

leads to the conversion of isofuranodiene into curzerene at high temperatures during GC-MS analysis [33].

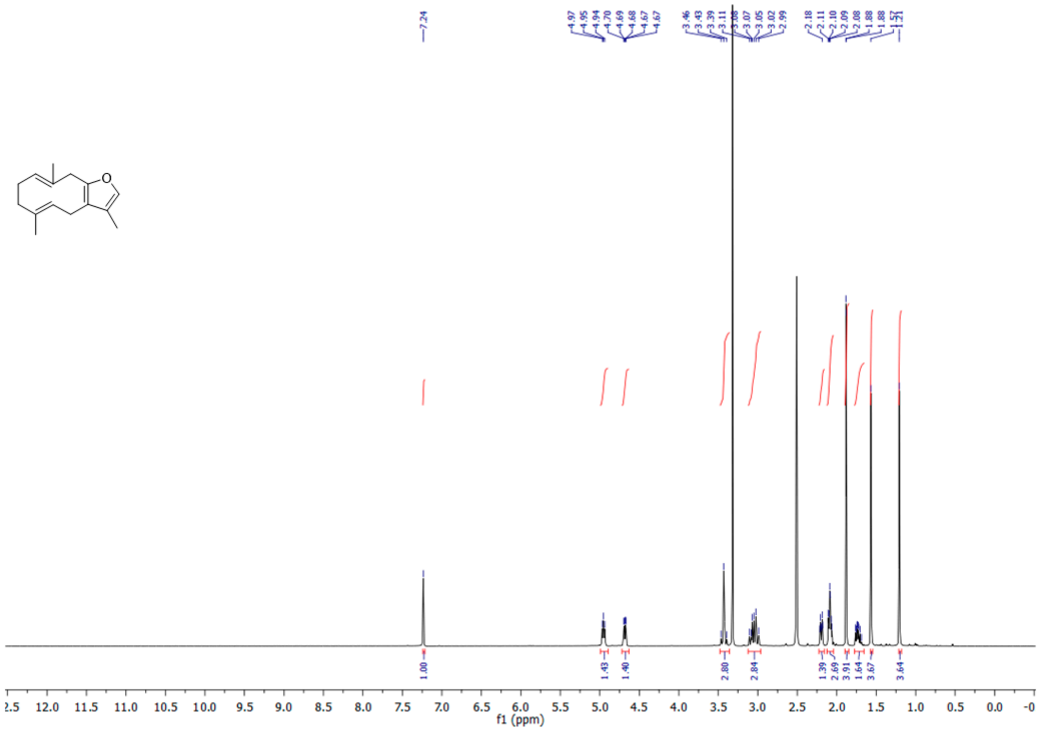

Figure S1. ^{1}H NMR spectrum of isofuranodiene.

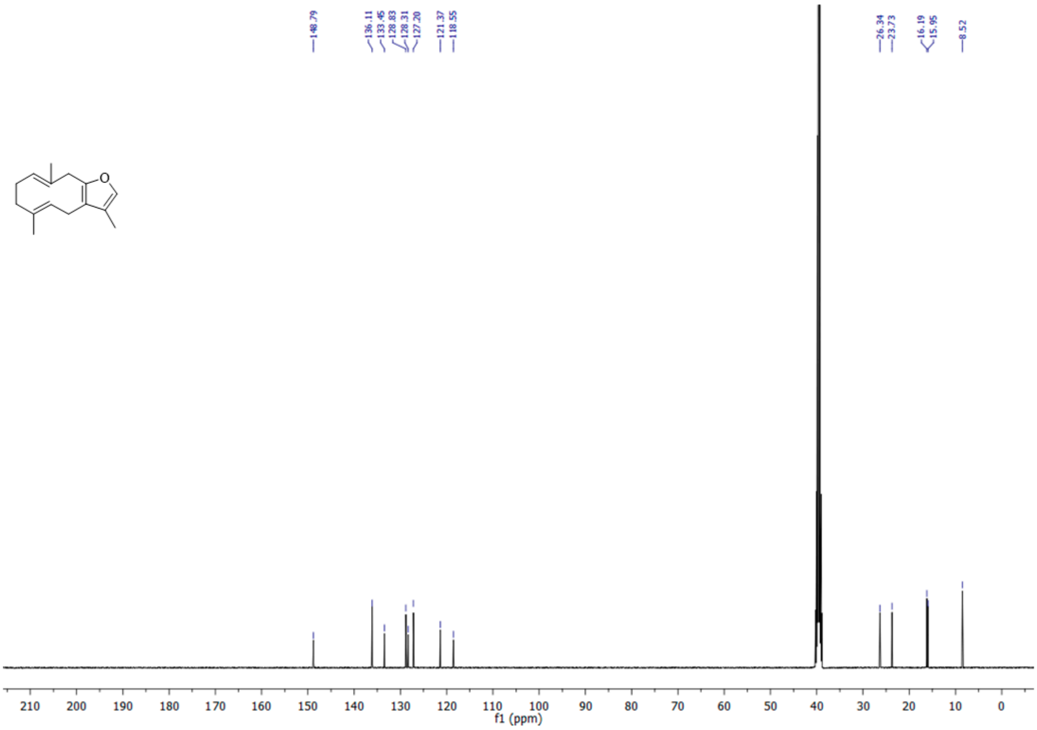

Figure S2. ^{13}C NMR spectrum of isofuranodiene.

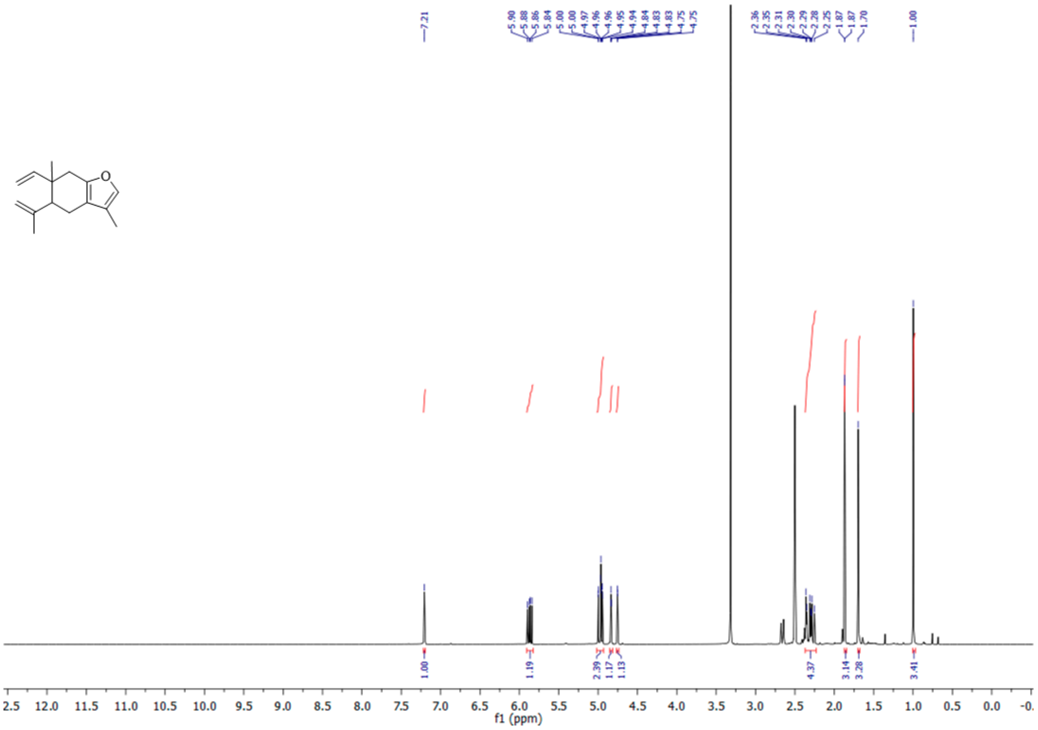

Figure S3. ^{1}H NMR spectrum of curzerene.

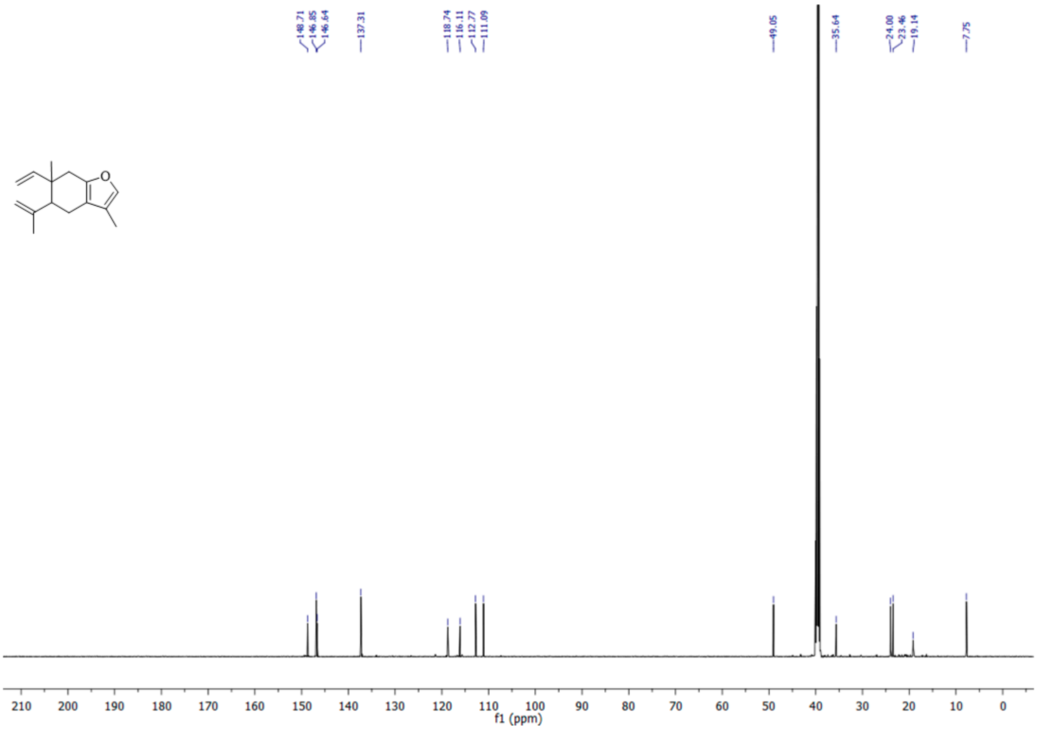

Figure S4. ^{13}C NMR spectrum of curzerene.

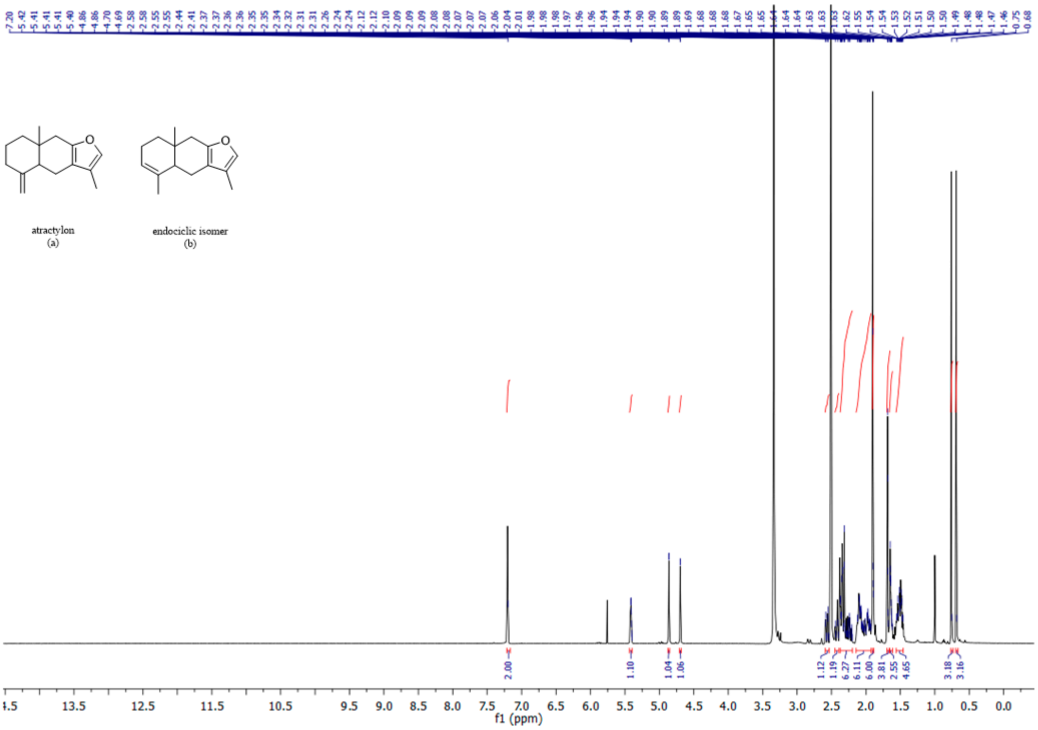

Figure S5. ^{1}H NMR spectrum of atractylon.

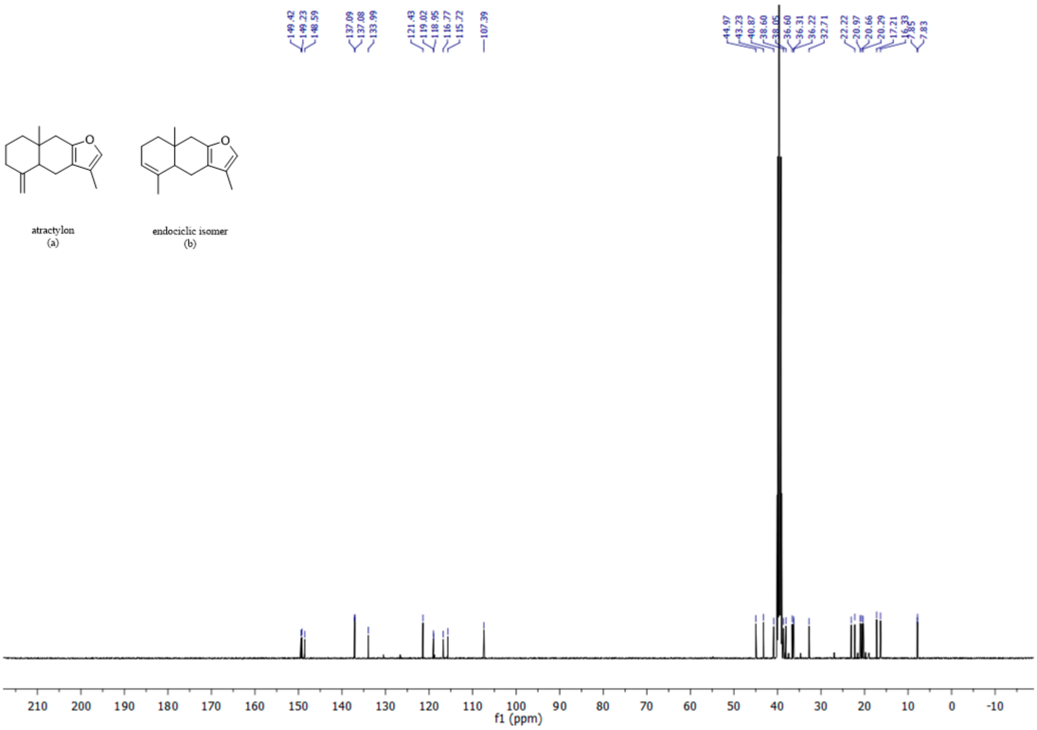

Figure S6. ^{13}C NMR spectrum of atractylon.

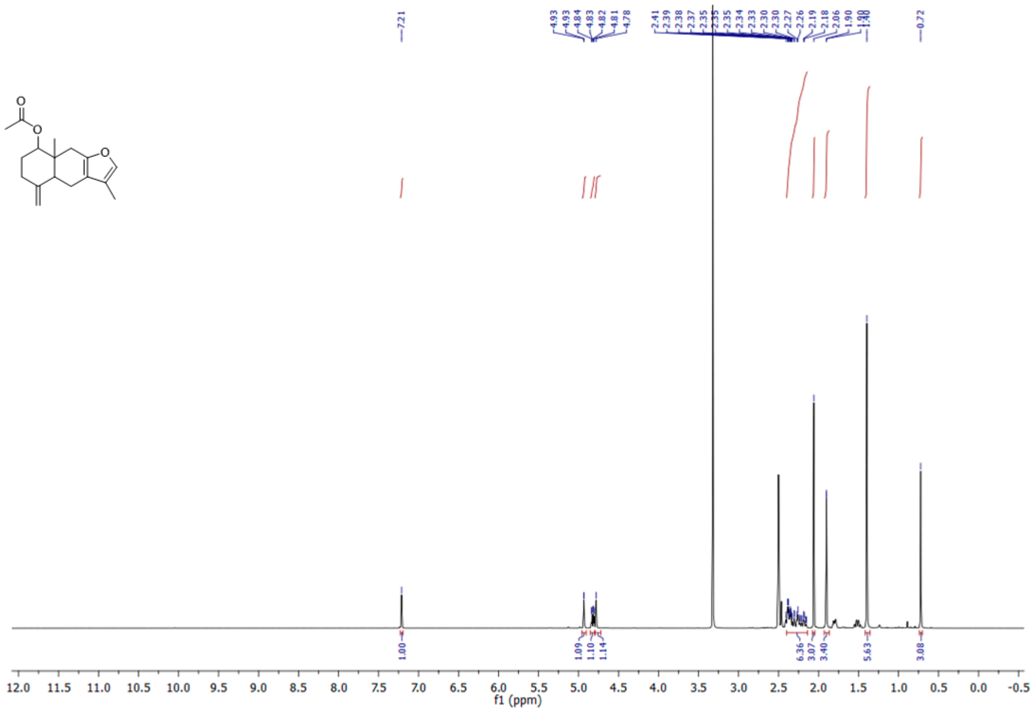

Figure S7. ^{1}H NMR spectrum of 1-β-acetoxyfuranoeudesm-4(15)-ene.

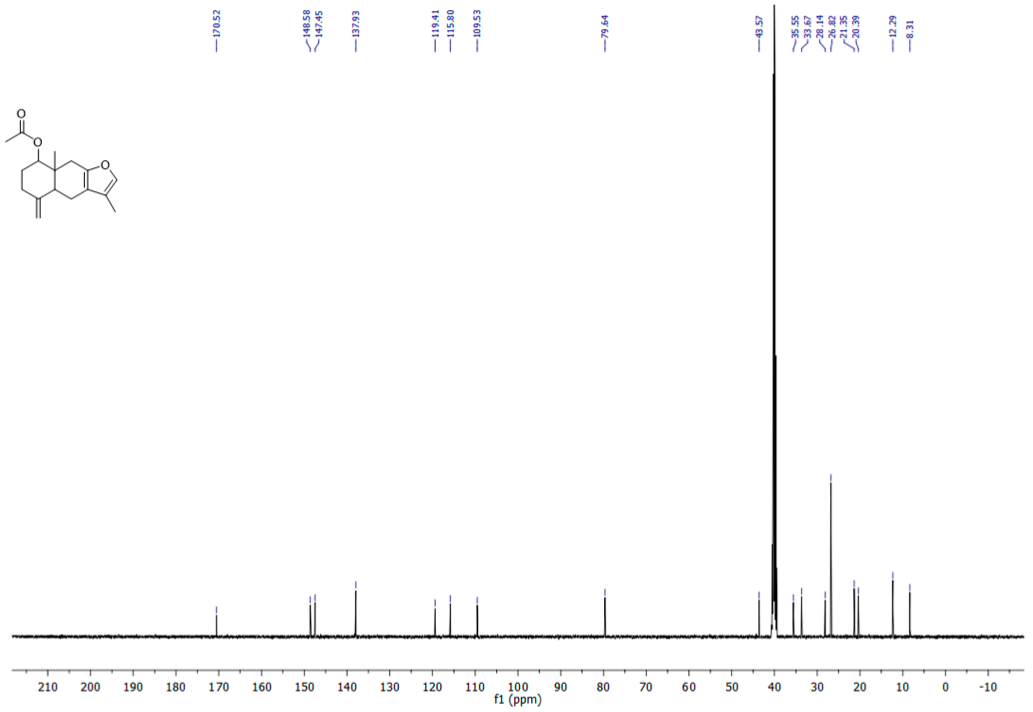

Figure S8. ^{13}C NMR spectrum of 1-β-acetoxyfuranoeudesm-4(15)-ene.

References

29. Van den Dool, H.; Kratz, P.D. A generalization of the retention index system including linear temperature programmed gas—liquid partition chromatography. *J. Chromatogr. A* **1963**, *11*, 463–471.
30. Adams, R. *Identification of essential oil components by gas chromatography/mass spectrometry*, 4th ed.; Allured Publishing Corp.: Carol Stream, IL, USA, 2007.
31. NIST, 2020. *Mass Spectral Library* (NIST/EPA/NIH). 17. National Institute of Standards and Technology: Gaithersburg, USA, 2020.
32. Papaioannou, F.: Koutsaviti, A.; Tzakou, O. Volatile constituents of different parts of *Smyrnium olusatrum* from Greece. *Nat. Prod. Commun.* **2010**, *5*(11), 1934578X1000501124. https://doi.org/10.1177/1934578X100050112
33. Maggi, F.; Papa, F.; Giuliani, C.; Maleci Bini, L.; Venditti, A.; Bianco, A.; Nicoletti, M.; Iannarelli, R.; Caprioli, G.; Sagratini, G.; Cortese, M.; Ricciutelli M.; Vittori, S. Essential oil chemotypification and secretory structures of the neglected vegetable *Smyrnium olusatrum* L. (Apiaceae) growing in central Italy. *Flavour Frag. J.* **2015**, *30*(2), 139-159. https://doi.org/10.1002/ffj.3221
